# Clinical Significance of Overlap Syndrome of Histologically Confirmed Lupus Nephritis with Antineutrophil Cytoplasmic Antibody-Associated Vasculitis

**DOI:** 10.3390/jcm13195831

**Published:** 2024-09-29

**Authors:** Jeong Yeop Whang, Jang Woo Ha, Yong-Beom Park, Sang-Won Lee

**Affiliations:** 1 Department of Medicine, Yonsei University College of Medicine, Seoul 03722, Republic of Korea; jywhang03@naver.com; 2 Division of Rheumatology, Department of Internal Medicine, Yonsei University College of Medicine, Seoul 03722, Republic of Korea; hjwnmk@yuhs.ac (J.W.H.); yongbpark@yuhs.ac (Y.-B.P.); 3 Institute for Immunology and Immunological Diseases, Yonsei University College of Medicine, Seoul 03722, Republic of Korea

**Keywords:** overlap syndrome, lupus nephritis, antineutrophil cytoplasmic antibody, vasculitis, contributor

## Abstract

**Objectives:** We applied the 2022 American College of Rheumatology/ European Alliance of Association for Rheumatology (ACR/EULAR) criteria for antineutrophil cytoplasmic antibody (ANCA)-associated vasculitis (AAV) to patients histologically diagnosed with lupus nephritis (LN) to investigate the overall rate of and initial contributing factors to the reclassification of overlap syndrome of LN with AAV (OS-LN-AAV). **Methods:** We retrospectively reviewed the medical records of 1292 patients with systemic lupus erythematosus (SLE) and included 164 patients with LN in this study. Patient demographics, SLE manifestations, LN classes, and laboratory data, including ANCA levels, were recorded. All-cause mortality and end-stage kidney disease (ESKD) were evaluated as poor outcomes. **Results:** The median age of the 164 patients was 37.0 years, and 12.2% were men. The overall reclassification rate was 37.8%, of which 34.1% and 3.7% of the patients were reclassified as having OS-LN-microscopic polyangiitis and OS-LN-granulomatosis with polyangiitis (GPA), respectively, but none as having eosinophilic GPA. ANCA positivity and AAV-suggesting lung lesions were major contributors to OS-LN-AAV reclassification. When patients were compared based on OS-LN AAV reclassification, ANCA positivity and myeloperoxidase-ANCA (or P-ANCA) positivity favoured for OS-LN-AAV reclassification, whereas oral ulcers did not. However, OS-LN-AAV reclassification did not affect all-cause mortality or ESKD. **Conclusions:** This is the first study demonstrating a 37.8% reclassification rate in patients histologically diagnosed with LN using the 2022 ACR/EULAR criteria for AAV. Furthermore, it was also the first to reveal ANCA positivity and AAV-suggesting lung lesions as major contributors to OS-LN-AAV reclassification.

## 1. Introduction

Systemic lupus erythematosus (SLE) is a chronic autoimmune disease characterised by variable systemic clinical manifestations influenced by genetic, epigenetic, environmental, and immunomodulatory factors [1,2]. Lupus nephritis (LN) is one of the most serious manifestations of SLE, affecting approximately 50% of SLE patients, with approximately 10% of them progressing to end-stage kidney disease (ESKD) requiring renal replacement therapy, which leads to higher mortality and lower life expectancy [3,4,5]. LN is classified into six classes based on the histological findings as follows: I, minimal mesangial LN; II, mesangial proliferative LN; III, focal or global endocapillary or extra-capillary glomerulonephritis (GN) involving < 50% of all glomeruli; IV, similar to class III but ≥50% of all glomeruli; V, membranous LN; and VI, advanced sclerotic LN. Among these, classes III and IV show relatively rapid progression and poor prognosis and, thus, require aggressive and active treatment using conventional and newly developed therapeutic regimens to prevent progression to ESKD [6,7].

Antineutrophil cytoplasmic antibodies (ANCAs) are a group of antibodies that recognise antigens, including myeloperoxidase (MPO) and proteinase 3 (PR3), that are located within the cytoplasm of neutrophils. Once a pro-inflammatory microenvironment is created owing to various inflammatory mediators, circulating MPO-ANCAs and PR3-ANCAs may bind to primed neutrophils and induce ANCA-mediated neutrophil activation. Activated neutrophils may migrate beyond vessels and cause inflammation in adjacent tissue through neutrophil degranulation, reactive oxygen species production, and complement pathway activation, which may form a vicious cycle of inflammation [8,9,10,11]. These sequential inflammation-chain reactions may eventually initiate and accelerate ANCA-associated vasculitis (AAV), including microscopic polyangiitis (MPA), granulomatosis with polyangiitis (GPA), and eosinophilic GPA (EGPA), which are subclassified based on their clinical features [12,13]. Among the three AAV subtypes, MPA is predominantly linked to kidney involvement, with over 80–90% of patients exhibiting renal manifestations, of which approximately 65% display rapidly progressive glomerulonephritis [14,15]. Additionally, kidney involvement has been reported to be observed in 50–80% of GPA patients and in up to 50% of ANCA-positive EGPA patients, albeit at lower rates than those with patients with MPA [16].

Despite both LN and AAV–kidney involvement being classified as small-vessel vasculitis, a critical difference lies in the renal histological findings between them [12]. LN is a representative immune complex vasculitis characterised by immune complex deposition in glomeruli, whereas AAV–kidney involvement is a typical pauci-immune vasculitis that is characterised by no or few immune complexes in glomeruli [17,18,19]. Therefore, LN and AAV–kidney involvement are considered independent diseases that seldom coexist. Nevertheless, ANCA positivity was reported to be associated with high activity and chronicity indices in LN [20,21], suggesting that circulating ANCAs may play an important role in the pathogenesis of LN and that AAV–kidney involvement may coexist with LN in a limited subset.

Recently, a joint group of the American College of Rheumatology (ACR) and the European Alliance of Association for Rheumatology (EULAR) proposed the new classification criteria for MPA, GPA, and EGPA in 2022 (the 2022 ACR/EULAR criteria for AAV) [22,23,24]. In contrast to the previous criteria [12,13], the 2022 ACR/EULAR criteria have three noticeable changes concerning AAV–kidney involvement. First, kidney involvement is recognised only when pauci-immune GN is identified through a kidney biopsy. Second, different scores are assigned to pauci-immune items based on the AAV subtypes as follows: +3 scores to MPA, +1 score to GPA, no score to EGPA. Third, increased weighted scores are given to ANCA positivity [25]. Furthermore, other factors, such as newly proposed pulmonary fibrosis and interstitial lung disease (ILD) for MPA or underappreciated granulomatosis on biopsy for GPA, may have additional influence on the classification of AAV beyond kidney-related consideration [22,23,24].

While several case series have explored overlap syndrome of LN (OS-LN-AAV) with AAV or OS-SLE-AAV, no study has investigated the frequency of and the contributing factors to the reclassification of OS-LN-AAV in a considerable number of patients with LN [26,27,28]. Hence, in the present study, we applied the 2022 ACR/EULAR criteria for AAV to patients histologically diagnosed with LN and investigated the overall rate of and initial major contributing factors to OS-LN-AAV reclassification.

## 2. Materials and Methods

### 2.1. Study Population

**Figure 1:**
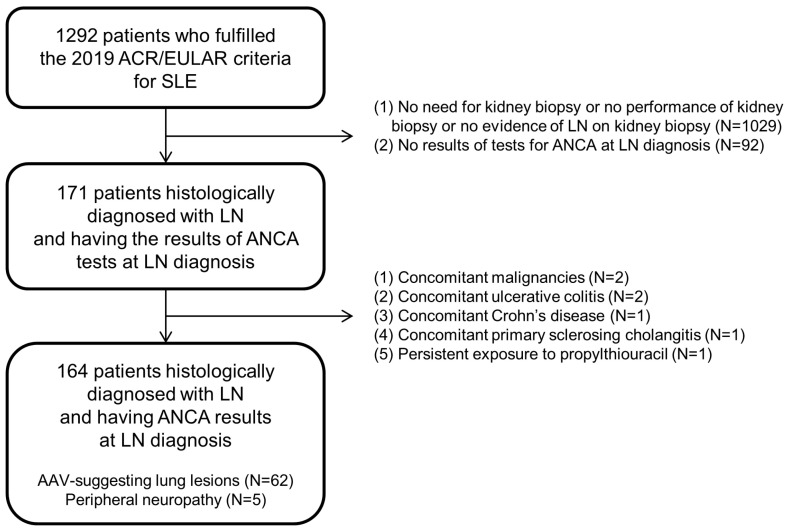
Algorithm of patient selection. ACR: the American College of Rheumatology; EULAR: the European Alliance Association of Rheumatology; LN: lupus nephritis; ANCA: antineutrophil cytoplasmic antibody; AAV: ANCA-associated vasculitis.

We retrospectively reviewed the medical records of 1292 patients who were diagnosed with SLE at the Division of Rheumatology, Department of Internal Medicine, Yonsei University College of Medicine, Severance Hospital, from March 2005 to December 2022. The inclusion criteria were as follows: (i) patients who were diagnosed with SLE at this hospital; (ii) patients who fulfilled the 2019 ACR/EULAR criteria for SLE [29]; (iii) patients who were histologically diagnosed with LN on kidney biopsy according to the International Society of Nephrology/Renal Pathology Society (ISN/RPS) 2003 classification of LN [30]; (iv) patients who had well-documented medical records sufficient for collecting clinical data at LN diagnosis or during follow-up; (v) patients who particularly had the results of ANCA tests performed at LN diagnosis; (vi) patients who did not have concomitant malignancies or serious infectious diseases [31,32]; (vii) patients who did not have concomitant autoimmune diseases affecting ANCA false positivity, for instance, Crohn’s disease, ulcerative colitis, or primary sclerosing cholangitis [33]; and (viii) patients who had never been exposed to drugs affecting ANCA false positivity, such as propylthiouracil [34]. Among the 1292 patients with SLE, 1029 patients were excluded owing to a lack of kidney biopsy requirements, lack of kidney biopsy, or no evidence of LN on kidney biopsy, and 92 were further excluded owing to a lack of ANCA test results obtained at LN diagnosis. Among the 171 patients histologically diagnosed with LN and having ANCA test results obtained at LN diagnosis, 2, 2, 1, 1, and 1 were excluded from this study owing to concomitant malignancies, ulcerative colitis, Crohn’s disease, primary sclerosing cholangitis, and persistent administration of propylthiouracil, respectively. Finally, 164 patients histologically diagnosed with LN were included in this study. Among the 164 patients, 62 and 5 had AAV-suggesting lung lesions and peripheral neuropathy, respectively, constituting a part of the signs suggestive of small- and medium-vessel vasculitis (Figure 1).

The present study was approved by the institutional review board (IRB) of Severance Hospital (Seoul, Korea; IRB No. 4-2023-1375). Given the retrospective design of the study and the use of anonymised patient data, the requirement for written informed consent was waived by the IRB.

### 2.2. Clinical Data at LN Diagnosis

In this study, signs suggestive of small- and medium-vessel vasculitis other than GN (LN) included two common clinical manifestations, AAV-suggesting lung lesions and peripheral neuropathy according to the 2022 ACR/EULAR criteria for AAV. AAV-suggesting lung lesion included two items: an item of pulmonary fibrosis or ILD for MPA and pulmonary nodules, mass, or cavitation for GPA. These lung lesions were confirmed through high-resolution computed tomography of the lungs. Peripheral neuropathy included all peripheral nervous system (PNS)-related symptoms and were confirmed through nerve conduction velocity studies [22,23,24]. Patient demographic data, including their age and sex, were obtained, and SLE manifestations and LN classes confirmed through kidney biopsy were recorded as SLE-specific data. Additionally, data on SLE-specific autoantibodies, complement levels (C3 and C4), and laboratory results were also collected [29]. In this study, perinuclear (P)-ANCAs and cytoplasmic (C)-ANCAs detected using an indirect immunofluorescence assay were approved as the ANCA results, along with MPO-ANCAs and PR3-ANCAs measured using an immunoassay according to the 2022 ACR/EULAR criteria for AAV, although an immunoassay has been recommended as the first line method for detecting ANCAs [22,23,24,35].

### 2.3. Clinical Data during Follow-Up

All-cause mortality and ESKD were considered poor outcomes in this study. The follow-up duration based on each poor outcome was defined as the period from LN diagnosis to the occurrence of the corresponding outcome for patients experiencing that particular corresponding outcome. Conversely, for those without the corresponding poor outcome, the follow-up duration was defined as the period from LN diagnosis to the last visit to the hospital. Additionally, the numbers of patients who had received drugs for SLE treatment during follow-up were recorded.

### 2.4. 2022 ACR/EULAR Classification Criteria for AAV

There are two mandatory requirements to apply the ACR/EULAR classification criteria for AAV: the presence of evidence of small- and medium-vessel vasculitis and the absence of concomitant serious medical conditions mimicking AAV. The differently weighted scores are assigned to each item of the 2022 ACR/EULAR criteria for MPA, GPA, and EGPA. When a total score is ≥5, MPA and GPA could be classified, whereas a total score ≥6 is required for the classification of EGPA [22,23,24].

### 2.5. Statistical Analyses

All statistical analyses were performed using SPSS Statistics for Windows, version 26 (IBM Corp., Armonk, NY, USA). Continuous and categorical variables are expressed as medians (interquartile ranges) and numbers (percentages), respectively. Significant differences between the two categorical and continuous variables were compared using the chi-square and Fisher’s exact tests and the Mann–Whitney U test, respectively. The odds ratio (OR) was obtained using the multivariable logistic regression analysis, including variables with *p* < 0.05 in the univariable logistic regression analysis. The comparison of the cumulative survival rates between the two groups was analysed using the Kaplan–Meier survival method with the log-rank test. A *p*-value < 0.05 was considered statically significant.

## 3. Results

### 3.1. Characteristics of Patients at LN Diagnosis

Among the 164 patients with LN, 62 (37.8%) had AAV-suggesting lung lesions. Among them, 32 (19.5%) exhibited pulmonary fibrosis or ILD, anticipating MPA, and 43 (26.2%) showed pulmonary nodules, mass, or cavitation on chest imaging, supporting GPA. The median age of the patients was 37.0 years, and 12.2% of them were men. In terms of SLE manifestations, the most common sign and symptom was proteinuria > 0.5 g/24 h (83.5%), followed by leukopaenia (60.4%), joint involvement (47.6%), and thrombocytopaenia (43.9%). Upon kidney biopsy, 101, 30, and 19 of the 164 patients were categorised as having class II or V, class III or IV, and class III or IV + V (mixed type), respectively. Additionally, fourteen patients exhibited other histological features, of which eight had class I, one had class VI, and five showed an unclassifiable class that was consistent with LN. ANCAs were positive in 61 (37.2%) patients, among whom 60 (36.6%) and 2 (1.2%) had MPO-ANCAs (or P-ANCAs) and PR3-ANCAs (or C-ANCAs), respectively. One patient had both types of ANCAs (Table 1).

### 3.2. Characteristics of Patients during Follow-Up

Among the 164 patients with LN, 7 (4.3%) died at a median follow-up duration of 77.5 months. Additionally, eight (4.9%) patients experienced progression to ESRD at a median follow-up duration of 14.0 months. The most commonly administered drug was glucocorticoids (95.1%), followed by mycophenolate mofetil (81.7%) and hydroxychloroquine (72.6%) (Table 1).

### 3.3. Application of the 2022 ACR/EULAR Criteria for MPA to LN Patients

In terms of clinical criteria, seven (4.3%) patients exhibited nasal involvement and received a score of -3. In terms of laboratory, imaging, and biopsy criteria, 60 (36.6%) and 32 (19.5%) patients exhibited MPO-ANCA (or P-ANCA) positivity (+6) and pulmonary fibrosis or ILD (+3), respectively. Pauci-immune GN was not identified upon kidney biopsy. Additionally, PR3-ANCAs (or C-ANCAs) (−1) were detected in two (1.2%) patients. Finally, given the AAV-suggesting lung lesions (pulmonary fibrosis or ILD) as signs suggestive of small- and medium-vessel vasculitis, 56 (34.1%) patients were reclassified as having OS-LN-MPA (Table 2).

### 3.4. Application of the 2022 ACR/EULAR Criteria for GPA to LN Patients

In terms of clinical criteria, seven (4.3%), three (1.8%), and nine (5.5%) patients received scores of +3, +2, and +1 owing to nasal involvement, cartilaginous involvement, and conductive or sensorineural hearing loss, respectively. In terms of laboratory, imaging, and biopsy criteria, 2 (1.2%) patients exhibited PR3-ANCAs (or C-ANCAs) (+5), 43 (26.2%) exhibited pulmonary nodules (+2), and 6 (3.7%) showed granulomatosis upon biopsy (+2). Paranasal sinusitis (+1) was found in 14 (18.5%) patients, but pauci-immune GN on kidney biopsy was not observed. Finally, given the AAV-suggesting lung lesions (pulmonary nodules) as signs suggestive of small- and medium-vessel vasculitis, six (3.7%) patients were reclassified as having OS-LN-GPA (Table 3).

### 3.5. Application of the 2022 ACR/EULAR Criteria for EGPA to LN Patients

In terms of clinical criteria, 21 (12.8%) patients received a score of +3 owing to obstructive airway disease. In terms of laboratory, imaging, and biopsy criteria, two (1.2%) patients exhibited extravascular eosinophilic-predominant inflammation upon biopsy (+2); however, peripheral eosinophilia was not detected. Among the 164 patients, 122 (74.4%) showed haematuria and lost one point. In particular, five patients exhibited peripheral neuropathy, which, however, did not turn out to be mononeuritis multiplex upon a nerve conduction velocity study. Accordingly, when considering GN as a consequence of LN and summating the scores received, no patient achieved a total score ≥ 6, and thus, none were reclassified as having OS-LN-EGPA (Table 4).

### 3.6. Comparison of the Variables between Patients with OS-LN-AAV and Those with LN but without AAV According to the 2022ACR/EULAR Criteria for AAV at LN Diagnosis and during Follow-Up

At the time of LN diagnosis, no significant differences in signs suggestive of small- and medium-vessel vasculitis, demographic data, LN types, and SLE-specific autoantibodies and complement levels were observed between patients with and those without OS-LN-AAV. Among SLE manifestations, compared to patients with LN but without AAV, those with OS-LN-AAV exhibited higher frequencies of leukopaenia (71.0% vs. 53.9%, *p* = 0.030) and pleural or pericardial effusion (43.5% vs. 27.5%, *p* = 0.034) but had a lower frequency of oral ulcers (3.2% vs. 14.7%, *p* = 0.019). MPO-ANCAs (or P-ANCAs) were detected in patients with OS-LN-AAV more frequently than those without (90.3% vs. 3.9%, *p* < 0.001), although there was no difference in the detection rates for PR3-ANCAs (or C-ANCAs). Additionally, patients with OS-LN-AAV showed significantly lower haemoglobin (10.3 g/dL vs. 11.2 g/dL, *p* = 0.032) and higher blood urea nitrogen levels (18.2 mg/dL vs. 14.6 mg/dL, *p* = 0.025) than those without OS-LN-AAV. During follow-up, no significant differences in poor outcomes and medications administered were found between the two groups except for methotrexate, which had been administered to patients with LN but without AAV; however, no clinical significance was noted (Table 5).

### 3.7. Multivariable Logistic Regression Analysis

To identify major contributing factors at LN diagnosis to OS-LN-AAV, we conducted the multivariable logistic regression analysis of the variables at LN diagnosis with statistical significance in the comparative analysis for OS-LN-AAV. Although both ANCA positivity and MPO-ANCA (or P-ANCA) positivity were significantly different between patients with OS-LN-AAV and those without, each of them was included in the corresponding logistic regression analysis. When ANCA positivity was included, oral ulcers (OR 0.050, 95% confidence interval [CI] 0.003, 0.797) were inversely associated with OS-LN-AAV, whereas ANCA positivity (OR 464.109, 95% CI 81.332, 2648.361) was positively associated with OS-LN-AAV. Additionally, when MPO-ANCA (or P-ANCA) positivity was included, similarly, oral ulcers (OR 0.066 95% CI 0.005, 0.920) and MPO-ANCA (or P-ANCA) positivity (OR 328.921, 95% CI 66.231, 1633.498) were significantly associated with OS-LN-AAV as well (Table S1).

### 3.8. Comparison of Cumulative Survival Rates

We compared cumulative patients’ and ESKD-free survival rates between the two groups according to OS-LN-AAV reclassification, ANCA positivity, and MPO-ANCA (or P-ANCA) positivity but found no significant differences at all (Figure S1).

## 4. Discussion

The background of planning and initiating this study was that we occasionally encountered clinical situations when we should have applied the 2022 ACR/EULAR criteria for AAV to patients suspected of both SLE and AAV at the same time in real clinical practice. That is, we had a question as to how to classify and establish therapeutic strategies among patients suspected of having undiagnosed SLE and AAV who met both criteria for the classification of SLE and AAV. Particularly, in usual clinical settings, if we encounter patients who were diagnosed with SLE by histologically confirmed LN and satisfied the 2022 ACR/EULAR criteria for AAV simultaneously, we thought that it would be difficult to make a clear decision on whether to classify these patients as having LN and stop the further diagnostic processes or to classify them as having OS-LN-AAV. Therefore, although this study was designed and conducted as a retrospective study, we thought it would be a clinically meaningful study to reveal the proportion of patients who could be classified as having OS-LN-AAV by applying the 2022 ACR/EULAR criteria for AAV to patients with histologically confirmed LN. Additionally, in the present study, we intended to deliver the message that the possibility of OS-LN-AAV should be considered rather than claim that those patients should be classified as having OS-LN-AAV.

On the other hand, despite the background of this study, when applying the 2022 ACR/EULAR criteria for AAV to SLE patients with histologically confirmed LN, two entry criteria in the 2022 ACR/EULAR criteria for AAV should be met. Especially, among the two criteria, one of “small-vessel vasculitis or medium-sized arteritis” is likely to be currently considered more important. In actual clinical practice, patients to whom the 2022 ACR/EULAR criteria for AAV can be applied must have symptoms of lung, kidney, and peripheral nerve involvements suggestive of AAV when excluding ANCA positivity and the allergic components. Because this study included patients with LN, the inclusion criteria included pulmonary and peripheral nervous systemic manifestations according to the 2022 ACR/EULAR criteria for AAV but not renal manifestations. Therefore, in this study, two entry criteria were sufficiently met and it would not be restricted to apply the 2022 ACR/EULAR criteria for AAV to SLE patients with histologically confirmed LN. Accordingly, this study has clinical implications in that it could provide an opportunity to reconsider the possibility of reclassification to OS-LN-AAV according to the newly proposed 2022 ACR/EULAR criteria for AAV in patients with histologically confirmed LN regardless of ANCA titres and positivity.

In this study, we applied the 2022 ACR/EULAR criteria for AAV to patients histologically diagnosed with LN and investigated the overall rate of and the initial contributing factors to OS-LN-AAV reclassification. We obtained several interesting findings. First, of the 164 patients histologically diagnosed with LN and having ANCA results at LN diagnosis, 62 (37.8%) were reclassified as having OS-LN-MPA and OS-LN-GPA but not having OS-ON-EGPA according to the 2022 ACR/EULAR criteria for AAV. Second, 56 (34.1%) patients were reclassified as having OS-LN-MPA, and MPO-ANCA (or P-ANCA) positivity and ILD at LN diagnosis evenly contributed to its reclassification. Third, six (3.7%) patients were reclassified as having OS-LN-GPA, and the contributing factors to its reclassification were mainly pulmonary nodules, followed by granulomatosis on biopsy and PR3-ANCA (or C-ANCA) positivity. In summary, ANCA positivity and AAV-suggesting lung lesions were identified as major contributing factors to OS-LN-AAV reclassification. Fourth, when clinical data at LN diagnosis were compared according to OS-LN-AAV reclassification, ANCA positivity and MPO-ANCA (or P-ANCA) positivity were proven to be favourable for OS-LN-AAV reclassification, whereas oral ulcers were against it. However, OS-LN-AAV reclassification was not associated with all-cause mortality or ESKD.

Whereas unlike traditional risk factors for ESKD such as serum blood urea nitrogen (HR 1.044, 95% confidence interval [CI] 1.024, 1.065) and creatinine levels (HR 0.897, 95% CI 0.398, 1.023), neither OS-LN-AAV nor ANCA positivity at LN diagnosis were significantly associated with ESKD during follow-up. Taken together with these results, we conclude that a third of patients histologically diagnosed with LN and having ANCA results at LN diagnosis were reclassified as having OS-LN-MPA or OS-LN-GPA, and both ANCA positivity and AAV-suggesting lung lesions primarily contributed to their reclassification. However, we could not find the predictors of ESKD during follow-up among OS-LN-AAV-related variables at LN diagnosis.

At the entry of this study, it was expected that patients with OS-LN-AAV exhibited a higher proportion of progression to ESKD during follow-up than those without OS-LN-AAV for the following reasons: The first assumption was regarding the link between ANCA positivity and the irreversible deterioration of kidney function. A previous study demonstrated that ANCA positivity was associated with the chronicity index among the histological findings of LN; however, this study failed to reveal that ANCA positivity at LN diagnosis was significantly associated with all-cause mortality or ESKD occurrence during follow-up [21], whereas another recent study reported that the chronicity indices were significantly associated with an increased proportion of progression to ESKD in patients with LN, which may indirectly prove the close link between ANCA positivity and the risk for progression to ESKD [36]. Therefore, it can be assumed that ANCA positivity as an aggravating factor in LN may predict an increase in the incidence of ESKD by reflecting irreversible damage.

The second assumption was regarding the possibility of the simultaneous LN and ANCA-associated GN, in other words, the coexisting two conflicting histological features of GN, immune-complex vs. pauci-immune vasculitis. Indeed, previous studies reported pauci-immune necrotising and crescentic glomerulonephritis not only in patients with LN but also in patients with SLE-specific antibodies as well as ANCAs [26,27,37]. Therefore, it can be assumed that the coexistence of ANCA-associated GN with LN may accelerate an increase in the incidence of ESKD compared to LN alone. However, this study could not demonstrate a significant increase in the proportion of progression to ESKD in patients reclassified as having OS-LN-AAV compared to those not having, and the additional histological analysis regarding the coexistence of both LN and ANCA-associated glomerulonephritis could not be performed due to the limitation of a retrospective study, whereas the significant association between blood urea nitrogen and serum creatinine levels with ESKD validated that there was no problem with the reliability of this result.

As was mentioned above, the poor renal prognosis was not determined by OS-LN-AAV reclassification or ANCA positivity, particularly MPO-ANCA (or P-ANCA) positivity. Otherwise, what is the clinical significance of OS-LN-AAV reclassification using clinical data at diagnosis? To answer this question, we focused on the presence of ILD, which contributed significantly to OS-LN-AAV reclassification, and further looked at the histological findings of granulomatosis, which contributed significantly to the reclassification of OS-LN-GPA. Firstly, in terms of ILD, among patients with AAV, it has been reported that ILD occurs in 13–14% of patients with AAV and 20% of MPO-ANCA-positive patients, a UPI pattern is observed in most cases (up to 75%), and the fibrotic type of ILD is significantly associated with a worse outcome, particularly an increase in the rate of all-cause mortality [38,39,40,41]. In contrast, among patients with SLE, it has been known that ILD occurs in 1.2–4% of patients with SLE, and similarly, it is associated with all-cause mortality; however, its severity is not noticeable compared to other pulmonary manifestations of SLE such as lupus pneumonitis or pulmonary arterial hypertension [42,43]. The difference in the frequency or severity of ILD between the two diseases is expected to result in differences of interest in the presence or future occurrence of ILD. Therefore, one clinical purpose of OS-LN-AAV reclassification is to reduce the risk of poor prognosis due to ILD by paying more attention to ILD and conducting careful follow-up.

Secondly, in terms of granulomatosis, among patients with SLE, chronic granulomatous inflammation (or disease) has been very rarely reported in case reports to date, unlike patients with GPA [44,45]. Of the six patients exhibiting histologic findings of granulomatous inflammation, only two were reclassified as having OS-LN-GPA. Although not all patients with granulomatosis were reclassified, the other clinical purpose of OS-LN-GPA reclassification is to drive the course of the disease toward good prognoses through close observations by performing serial GPA-specific laboratory and radiological tests. Additionally, we compared the cumulative patients’ and ESKD-free survival rates between patients according to the presence of ILD and granulomatous inflammation. Firstly, for ILD, patients with ILD tended to exhibit a lower cumulative patients’ survival rate than those without ILD, but no statistical significance was obtained (*p* = 0.093). Meanwhile, no difference in ESKD-free survival rates was observed between the two groups (Figure S2). Next, for granulomatous inflammation, because only 6 of the 164 patients showed granulomatous changes on biopsy, we found no significant results in the comparative analysis.

We would like to add our opinions on four issues: (i) Regarding anti-ds DNA/anti-Sm antibodies, they were detected in 17.7% of the patients in this study. Considering the contribution of those antibodies to the development and aggravation of LN predominantly [46], it could be speculated that they might have driven the disease pattern toward LN rather than AAV and further played a negative role in determining the reclassification of OS-LN-AAV in already-diagnosed LN patients. Hence, to clarify the role of the presence of anti-ds DNA/anti-Sm antibodies in reclassifying the study subjects as having OS-LN-AAV, we divided the patients into two groups according to the presence of anti-ds DNA/anti-Sm antibodies and compared the frequency of OS-LN-AAV between the two groups. Among the 135 patients with anti-ds DNA/anti-Sm antibodies, 53 (39.3%) were reclassified as having OS-NL-AAV, and 9 of the 29 patients (31.0%) without those antibodies were reclassified as having OS-NL-AAV. This result drew no significant statistical difference between the two groups. Therefore, it could be concluded that there was no influence of the presence of LN-specific antibodies on OS-LN-AAV reclassification. (ii) Given that both lupus vasculitis and AAV could induce inflammation in medium-sized arteries [12,47], it would be necessary to consider and check the possibility of renal artery involvement of both vasculitis types in the study population, particularly in those reclassified as having OS-LN-AAV. Although lupus vasculitis with renal artery involvement cannot directly cause glomerulonephritis (lupus nephritis), it is likely to provoke two significant forms of indirect renal damage. First, proximal renal arterial involvement has the potential to induce systemic hypertension and subsequent secondary hypertensive nephropathy via the renin–angiotensin–aldosterone system [48]. Second, hypo-perfusion to the kidneys owing to renal arterial thrombosis also has the potential to induce ischaemic nephropathy [49]. However, in this study, we found no patients exhibiting clinically relevant primary hypertensive nephropathy or secondary ischaemic nephropathy in both groups of patients with and without OS-LN-AAV. (iii) Compared to the previous study conducted by Turner-Stokes et al., the frequency of ANCA positivity in this study was relatively high [50]. The reason for the difference is that Turner-Stokes’s study included ANCA results via an immunoassay (MPO-ANCA and PR3-ANCA), whereas our study included not only ANCA results using an immunoassay but also those using an indirect immunofluorescence assay (P-ANCA and C-ANCA) based on the 2022 ACR/EULAR criteria for AAV [22,23,24,25]. However, similar to Turner’s study, our study also demonstrated that ANCA positivity did not affect the cumulative ESKD-free survival rates during the follow-up period regardless of OS-LN-AAV reclassification using Kaplan–Meier survival analysis with a log-rank test (*p* = 0.904). (iv) In this study, methotrexate was administered to only 10 patients with OS-LN-AAV. A detailed review of medical records revealed that all 10 patients receiving methotrexate had complained of arthralgia or arthritis. To clarify the association between methotrexate administration and OS-LN-AAV reclassification, we performed univariable logistic regression and obtained no significant odds ratio of methotrexate for OS-LN-AAV reclassification (*p* = 0.999).

The advantage of this study is that it was the first to apply the 2022 ACR/EULAR criteria for AAV to patients histologically diagnosed with LN and demonstrate the reclassification rate of OS-LN-AAV, OS-LN-MPA, and OS-LN-GPA as 37.8% and, furthermore, ANCA positivity and AAV-suggesting lung lesions as the contributing factors to their reclassification. In addition, another advantage is that it was the first to present the message that poor prognosis due to major organ involvement by AAV could be coped with by not excluding the possibility that AAV may coexist even in SLE patients histologically confirmed as LN.

This study has several limitations. The first drawback is that the number of participating patients in this study is relatively small due to it being a single-centre study. However, because patients in this study had been diagnosed with SLE by the same rheumatologists with a consensus on the same diagnostic classification criteria at the same institution, inter-centre and inter-observer variations may have been minimised. The second drawback is the limitation of data collection due to using the retrospective study method. Therefore, the reliability of the collected data might have been inevitably lower than that of prospective research methods. The most notable limitation is that it was not possible to confirm the presence of pauci-immune GN, which might be found to coexist with immune-complex GN by re-performing immunofluorescence staining, because frozen samples of kidney tissue from all patients included in this study had not been stored. If the 2022 ACR/EULAR criteria for AAV are applied to patients newly diagnosed as LN in many institutions and the renal tissue findings of patients who are reclassified as having OS-LN-AAV are rechecked, the relatively accurate proportion and pattern of OS-LN-AAV in patients histologically diagnosed with LN may be assessed. Additionally, given the mutual clinical features of the lungs and kidneys in both LN and AAV, it may be clinically important to elucidate the association between renal function and lung function deterioration. However, due to a very small number of patients undergoing pulmonary function tests and a too widely ranged follow-up duration to assess the alteration in renal function, we could not provide any clues about the impact of the two variables on all-cause mortality and progression to ESKD in this study. Finally, we applied the criteria for AAV to LN patients, but we could not apply the diagnostic criteria for SLE (LN) to AAV patients. Strictly speaking, the latter could provide an opportunity to validate the results of this study in a bidirectional way. In the future, we will conduct an additional study to confirm the presence or absence of OS by applying the diagnostic classification criteria for SLE to patients who are currently diagnosed with AAV.

## 5. Conclusions

This is the first study to demonstrate that the reclassification rate of OS-LN-AAV was 37.8% of patients histologically diagnosed with LN according to the 2022 ACR/EULAR criteria for AAV. Additionally, ANCA positivity and AAV-suggesting lung lesions were identified as major contributing factors to OS-LN-AAV reclassification.

## Figures and Tables

**Table 1:** Characteristics of patients histologically diagnosed with LN and having ANCA results (N = 164).

Variables	Values
At LN diagnosis	
Signs suggestive of small- or medium-vessel vasculitis (N, (%))	
Glomerulonephritis (LN) on kidney biopsy	164 (100)
AAV-suggesting lung lesions	62 (37.8)
Pulmonary fibrosis or ILD on chest imaging (MPA-suggesting lung lesions)	32 (19.5)
Pulmonary nodule, mass, and cavitation on chest imaging (GPA-suggesting lung lesions)	43 (26.2)
Both MPA- and GPA-suggesting lung lesions	13 (7.9)
Peripheral neuropathy	5 (3.0)
Mononeuritis multiplex	0 (0.0)
Demographic data	
Age (years)	37.0 (22.0)
Male sex (N, (%))	20 (12.2)
SLE manifestations (N, (%))	
Fever	69 (42.1)
Leukopaenia	99 (60.4)
Thrombocytopaenia	72 (43.9)
Autoimmune haemolysis	14 (8.5)
Delirium	1 (0.6)
Psychosis	0 (0)
Seizure	5 (3.0)
Non-scarring alopecia	6 (3.7)
Oral ulcers	17 (10.4)
Subacute cutaneous or discoid lupus	25 (15.2)
Acute cutaneous lupus	53 (32.3)
Pleural or pericardial effusion	55 (33.5)
Acute pericarditis	3 (1.8)
Joint involvement	78 (47.6)
Proteinuria > 0.5 g/24 h	137 (83.5)
LN classes on kidney biopsy (N, (%))	
Class II or V	30 (18.3)
Class III or IV	101 (61.6)
Class III or IV + V mixed type	19 (11.6)
Others *	14 (8.5)
Autoimmune antibodies and low C3 or C4 levels (N, (%))	
Positive antiphospholipid antibodies	59 (36.0)
Anti-DNA antibodies or anti-Smith antibodies	135 (82.3)
Low C3	115 (70.1)
Low C4	91 (55.5)
Laboratory results	
White blood cell count (/mm^3^)	4505.0 (3400)
Haemoglobin (g/dL)	10.7 (2.8)
Platelet count (×1000/mm^3^)	204.0 (124.2)
Blood urea nitrogen (mg/dL)	15.0 (11.5)
Serum creatinine (mg/dL)	0.8 (0.5)
Total protein (g/dL)	6.5 (1.5)
Serum albumin (g/dL)	3.2 (1.2)
Aspartate aminotransferase (IU/L)	25.5 (24.0)
Alanine aminotransferase (IU/L)	17.0 (17.0)
ESR (mm/h)	46.0 (54.0)
CRP (mg/L)	2.5 (9.1)
C3 (mg/dL)	50.1 (47.2)
C4 (mg/dL)	7.5 (12.8)
ANCA positivity (N, (%))	
MPO-ANCA (or P-ANCA) positivity	60 (36.6)
PR3-ANCA (or C-ANCA) positivity	2 (1.2)
ANCA positivity	61 (37.2)
During follow-up	
Poor outcomes	
All-cause mortality (N, (%))	7 (4.3)
Follow-up duration based on all-cause mortality (months)	77.5 (83.0)
ESKD (N, (%))	8 (4.9)
Follow-up duration based on EKRD (months)	14.0 (23.0)
Medications administered (N, (%))	
Total	163 (99.4)
Glucocorticoids	156 (95.1)
Hydroxychloroquine	119 (72.6)
Mycophenolate mofetil	134 (81.7)
Tacrolimus	44 (26.8)
Methotrexate	10 (6.1)
Cyclophosphamide	72 (43.9)

Values are expressed as a median (interquartile range, IQR) or N (%). * Others = class I (8), class VI (1), and unclassifiable but consistent with LN (5). LN: lupus nephritis; AAV: ANCA-associated vasculitis; ANCA: antineutrophil cytoplasmic antibody; ILD: interstitial lung disease; MPO: myeloperoxidase; P: perinuclear; PR3: proteinase 3; C: cytoplasmic; SLE: systemic lupus erythematosus; ESKD: end-stage kidney disease.

**Table 2:** Application of the 2022 ACR/EULAR criteria for MPA to LN patients (N = 164).

Variables		Values
At LN diagnosis	**Score**	**(N (%))**
Items for the 2022 ACR/EULAR criteria for MPA and assigned scores to each item		
Clinical criteria		
Nasal involvement (discharge, ulcers, crusting, congestion, and septal defect/perforation)	−3	7 (4.3)
Laboratory, imaging, and biopsy criteria		
MPO-ANCA (or P-ANCA) positivity	+6	60 (36.6)
Pulmonary fibrosis or ILD on chest imaging	+3	32 (19.5)
Pauci-immune glomerulonephritis on biopsy	+3	0 (0.0)
PR3-ANCA (or C-ANCA) positivity	−1	2 (1.2)
Serum eosinophil count ≥ 1000/μL	−4	0 (0.0)
Patients with a total score ≥ 5		56 (34.1)

ACR: American College of Rheumatology; EULAR: European Alliance of Associations for Rheumatology; MPA: microscopic polyangiitis; LN: lupus nephritis; MPO: myeloperoxidase; ANCA: antineutrophil cytoplasmic antibody; P: perinuclear; ILD: interstitial lung disease; PR3: proteinase 3; C: cytoplasmic.

**Table 3:** Application of the 2022 ACR/EULAR criteria for GPA to LN patients (N = 164).

Variables		Values
At LN diagnosis	**Scores**	**(N (%))**
Items for the 2022 ACR/EULAR criteria for GPA and assigned scores to each item		
Clinical criteria		
Nasal involvement (discharge, ulcers, crusting, congestion, and septal defect/perforation)	+3	7 (4.3)
Cartilaginous involvement	+2	3 (1.8)
Conductive or sensorineural hearing loss	+1	9 (5.5)
Laboratory, imaging, and biopsy criteria		
PR3-ANCA (or C-ANCA) positivity	+5	2 (1.2)
Pulmonary nodules, mass, or cavitation on chest imaging	+2	43 (26.2)
Granuloma, granulomatous inflammation, or giant cells on biopsy	+2	6 (3.7)
Nasal/paranasal sinusitis or mastoiditis on imaging	+1	14 (18.5)
Pauci-immune glomerulonephritis on biopsy	+1	0 (0.0)
MPO-ANCA (or P-ANCA) positivity	−1	60 (36.6)
Serum eosinophil count ≥ 1000/μL	−4	0 (0.0)
Patients with a total score ≥ 5 (N (%))		6 (3.7)

ACR: American College of Rheumatology; EULAR: European Alliance of Associations for Rheumatology; GPA: granulomatosis with polyangiitis; LN: lupus nephritis; PR3: proteinase 3; ANCA: antineutrophil cytoplasmic antibody; C: cytoplasmic; MPO: myeloperoxidase; P: perinuclear.

**Table 4:** Application of the 2022 ACR/EULAR criteria for EGPA to LN patients (N = 164).

Variables		Values
At LN diagnosis	**Score**	**(N (%))**
Items for the 2022 ACR/EULAR criteria for EGPA and assigned scores to each item		
Clinical criteria		
Obstructive airway disease	+3	21 (12.8)
Nasal polyps	+3	0 (0.0)
Mononeuritis multiplex	+1	0 (0.0)
Laboratory, imaging, and biopsy criteria		
Serum eosinophil count ≥ 1000/μL	+5	0 (0.0)
Extravascular eosinophilic-predominant inflammation on biopsy	+2	2 (1.2)
PR3-ANCA (or C-ANCA) positivity	−3	2 (1.2)
Haematuria	−1	122 (74.4)
Patients with a total score ≥ 6 (N (%))		0 (0.0)

ACR: American College of Rheumatology; EULAR: European Alliance of Associations for Rheumatology; EGPA: eosinophilic granulomatosis with polyangiitis LN: lupus nephritis; PR3: proteinase 3; ANCA: antineutrophil cytoplasmic antibody; C: cytoplasmic.

**Table 5:** Comparison of the variables between patients with OS-LN-AAV and those with LN but without AAV according to the 2022ACR/EULAR criteria for AAV.

Variables	Patients with OS-LN-AAV (N = 62)	Patients with LN but without AAV (N = 102)	*p*-Value
At the time of diagnosis			
Signs suggestive of small- or medium-vessel vasculitis (N, (%))			
Glomerulonephritis (LN)	62 (100)	102 (100)	N/A
AAV-suggesting lung lesions	23 (37.1)	39 (38.2)	0.884
Fibrosis or ILD on chest imaging	9 (14.5)	23(22.5)	0.208
Lung nodule, mass, and cavitation on chest imaging	17 (27.4)	26 (25.5)	0.785
Peripheral neuropathy	2 (3.2)	3 (2.9)	0.918
Mononeuritis multiplex	0 (0)	0 (0)	N/A
Demographic data			
Age (years)	40.0 (28.0)	36.5 (19.0)	0.076
Male gender (N, (%))	6 (9.7)	14 (13.7)	0.442
SLE manifestations (N, (%))			
Fever	22 (35.5)	47 (46.1)	0.183
Leukopaenia	44 (71.0)	55 (53.9)	0.030
Thrombocytopaenia	32 (51.6)	40 (39.2)	0.121
Autoimmune haemolysis	4 (6.5)	10 (9.8)	0.456
Delirium	0 (0.0)	1 (1.0)	0.434
Psychosis	0 (0.0)	0 (0)	N/A
Seizure	1(1.6)	4 (3.9)	0.404
Non-scarring alopecia	4 (6.5)	2 (2.0)	0.137
Oral ulcers	2 (3.2)	15 (14.7)	0.019
Subacute cutaneous or discoid lupus	6 (9.7)	19 (18.6)	0.122
Acute cutaneous lupus	16 (25.8)	37 (36.3)	0.165
Pleural or pericardial effusion	27 (43.5)	28 (27.5)	0.034
Acute pericarditis	1 (1.6)	2 (2.0)	0.872
Joint involvement	29 (46.8)	49 (48.0)	0.875
Proteinuria > 0.5 g/24 h	49 (79.0)	88 (86.3)	0.225
LN types on renal biopsy (N, (%))			
Class II or V	11 (17.7)	19 (18.6)	0.887
Class III or IV	41 (66.1)	60 (58.8)	0.351
Class III/IV + V mixed type	5 (8.1)	14 (13.7)	0.272
Others	5 (8.1)	9 (8.8)	0.866
Autoimmune antibodies and low C3 or C4 levels (N, (%))			
Positive antiphospholipid antibodies	22 (35.5)	37 (36.3)	0.919
Anti-DNA antibodies or anti-Smith antibodies	53 (85.5)	82 (80.4)	0.407
Low C3	39 (62.9)	76 (74.5)	0.115
Low C4	31 (50.0)	60 (58.8)	0.270
ANCA positivity (N, (%))			
MPO-ANCA (or P-ANCA) positivity	56 (90.3)	4 (3.9)	<0.001
PR3-ANCA (or C-ANCA) positivity	2 (3.2)	0 (0)	0.068
ANCA positivity	57 (91.9)	4 (3.9)	<0.001
Laboratory results			
White blood cell count (/mm^3^)	4270.0 (2660.0)	4720.0 (3715.0)	0.268
Haemoglobin (g/dL)	10.3 (2.4)	11.2 (2.8)	0.032
Platelet count (x1,000/mm^3^)	180.0 (123.0)	208.0 (115.0)	0.092
Blood urea nitrogen (mg/dL)	18.2 (12.7)	14.6 (8.8)	0.025
Serum creatinine (mg/dL)	0.8 (0.5)	0.7 (0.5)	0.419
Total protein (g/dL)	6.3 (1.5)	6.5 (9.3)	0.554
Serum albumin (g/dL)	3.0 (1.3)	3.2 (1.1)	0.190
Aspartate aminotransferase (IU/L)	26.5 (19.0)	25.0 (25.0)	0.532
Alanine aminotransferase (IU/L)	17.0 (10.0)	16.5 (24.0)	0.251
ESR (mm/hr)	45.0 (61.0)	49.0 (52.0)	0.819
CRP (mg/L)	2.8 (11.5)	2.3 (6.8)	0.333
C3 (mg/dL)	47.7 (41.6)	52.8 (48.4)	0.336
C4 (mg/dL)	5.0 (10.9)	8.4 (13.8)	0.217
During follow-up			
Poor outcomes			
ESRD (N, (%))	2 (3.2)	6 (5.9)	0.444
Follow-up duration based on ESRD (months)	57.5 (63.0)	93.5 (98.0)	0.001
CVA (N, (%))	4 (6.5)	3 (2.9)	0.281
Follow-up duration based on CVA (months)	54.5 (60.0)	93.5 (99.0)	0.000
ACS (N, (%))	1 (1.6)	3 (2.9)	0.593
Follow-up duration based on ACS (months)	57.5 (63.0)	93.5 (98.0)	0.000
All-cause mortality (N, (%))	1 (1.6)	6 (5.9)	0.190
Follow-up duration based on all-cause mortality (months)	57.5 (60.0)	94.5 (95.0)	0.000
Medications administered (N, (%))			
Total	61 (98.4)	102 (100)	0.198
Glucocorticoids	60 (96.8)	96 (94.1)	0.444
Hydroxychloroquine	48 (77.4)	71 (69.6)	0.277
Mycophenolate mofetil	50 (80.6)	84 (82.4)	0.784
Tacrolimus	16 (25.8)	28 (27.5)	0.818
Methotrexate	0 (0)	10 (9.8)	0.011
Cyclophosphamide	32 (51.6)	40 (39.2)	0.121

Values are expressed as a median (interquartile range, IQR) or N (%). LN: lupus nephritis; AAV: ANCA-associated vasculitis; ANCA: antineutrophil cytoplasmic antibody; ILD: interstitial lung disease; MPO: myeloperoxidase; P: perinuclear; PR3: proteinase 3; C: cytoplasmic; SLE: systemic lupus erythematosus; ESRD: end-stage renal disease; CVA: cerebrovascular accident; ACS: acute coronary syndrome.

## Data Availability

The data used to support the findings of this study are included within the article and the Supplementary Materials.

## Supplementary Materials

The following supporting information can be downloaded at: https://www.mdpi.com/article/10.3390/jcm13195831/s1, Table S1: Logistic regression of the variables at LN diagnosis with statistical significance in the comparative analysis for OS-LN-AAV; Figure S1: Comparative analysis of cumulative patients’ and ESKD-free survival rates according to the presence of OS-LN-AAV, ANCA positivity, and MPO-ANCA (or P-ANCA) positivity; Figure S2: Comparative analysis of cumulative patients’ and ESKD-free survival rates according to the presence of ILD; and Certificate for English Editing.
