# Supplementary material for: Clinical Significance of Overlap Syndrome of Histologically Confirmed Lupus Nephritis with Antineutrophil Cytoplasmic Antibody-Associated Vasculitis
Source: J Clin Med. 2024 Sep 29;13(19):5831. doi: 10.3390/jcm13195831 (PMC11478156; doi:10.3390/jcm13195831)
Supplement: Supplementary file 1 [file jcm-13-05831-s001.zip › jcm-3198852-supplementary.pdf]

Table S1. Logistic regression of the variables at LN diagnosis with statistical significance in the comparative analysis for OS-LN-AAV

Variables	Multivariable		
	**OR**	**95% CI**	**P value**
Including ANCA positivity			
Leukopaenia	1.460	0.323, 6.606	0.623
Oral ulcers	0.050	0.003, 0.797	0.034
Pleural or pericardial effusion	3.136	0.656, 14.999	0.152
ANCA positivity	464.109	81.332, 2648.361	<0.001
Haemoglobin	0.780	0.514, 1.186	0.246
Blood urea nitrogen	0.966	0.925, 1.008	0.110
Including MPO-ANCA (or P-ANCA)			
Leukopaenia	1.704	0.404, 7.177	0.468
Oral ulcers	0.066	0.005, 0.920	0.043
Pleural or pericardial effusion	2.346	0.546, 10.078	0.251
MPO-ANCA (or P-ANCA) positivity	328.921	66.231, 1633.498	<0.001
Haemoglobin	0.766	0.520, 1.127	0.176
Blood urea nitrogen	0.975	0.934, 1.018	0.255

LN: lupus nephritis; OS: overlap syndrome; AAV: ANCA-associated vasculitis; ANCA: antineutrophil cytoplasmic antibody; OR: odds ratio; CI: confidence interval; MPO: myeloperoxidase; P: perinuclear.

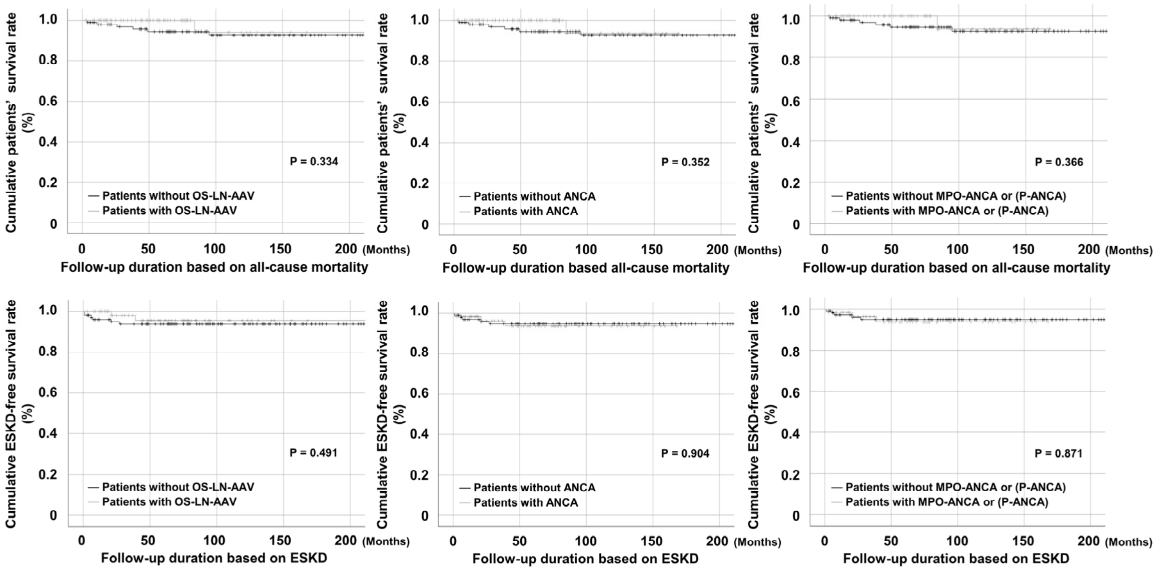

Figure S1: Comparative analysis of cumulative patients' and ESKD-free survival rates according to the presence of OS-LN-AAV, ANCA positivity, and MPO-ANCA (or P-ANCA) positivity.

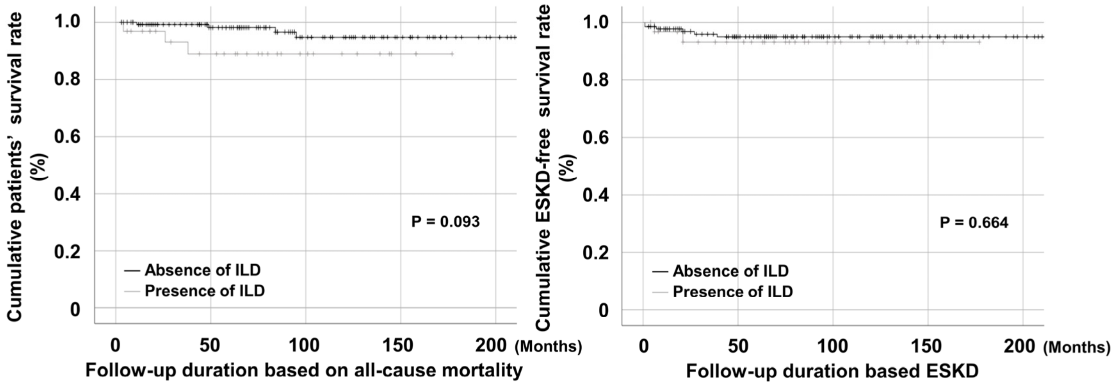

Figure S2: Comparative analysis of cumulative patients' and ESKD-free survival rates according to the pres-ence of ILD; and Certificate for English Editing.
